# Efficacy and safety of low dose rituximab in pemphigus: an updated systematic review and meta-analysis

**DOI:** 10.3389/fimmu.2025.1605243

**Published:** 2025-07-25

**Authors:** Si-Han Liu, Si-Hang Wang, Ya-Gang Zuo

**Affiliations:** Department of Dermatology, State Key Laboratory of Complex Severe and Rare Diseases, National Clinical Research Center for Dermatologic and Immunologic Diseases, Peking Union Medical College Hospital, Chinese Academy of Medical Sciences and Peking Union Medical College, Beijing, China

**Keywords:** meta-analysis, pemphigus, rituximab, safety, treatment outcome

## Abstract

**Objective:**

To evaluate the efficacy and safety of low-dose rituximab (RTX) in the treatment of pemphigus.

**Methods:**

A systematic literature search was conducted across PubMed, Embase, the Cochrane Library, and ClinicalTrials.gov to identify eligible studies. Primary efficacy outcomes included complete remission (CR), relapse rates, time to disease control (TDC), time to CR, and cumulative corticosteroid dose. Safety outcomes were assessed by meticulously documenting adverse events (AEs) and concomitant medications reported in each study.

**Results:**

The final analysis incorporated five comparative studies and nine single-arm studies investigating the efficacy and safety outcomes of low-dose RTX. Comparative data revealed no statistically significant differences between the high-dose and low-dose RTX groups in CR, relapse rates, TDC, time to CR, and cumulative corticosteroid dose. In single-arm studies, pooled CR and relapse rates were 63.2% and 28.6%, respectively. No fatal events were reported; however, severe AEs, including pneumonia and sepsis, were documented in the low-dose RTX cohort.

**Conclusion:**

Low-dose RTX exhibited comparable clinical efficacy to high-dose RTX regimens in pemphigus management. However, clinicians should remain vigilant for potential AEs associated with low-dose RTX infusion.

## 1 Introduction

Pemphigus is a group of rare, recurrent, and potentially life-threatening dermatological disorders characterized by flaccid blisters and erosions of the skin and/or mucous membranes. The pathogenesis of pemphigus is attributed to the abnormal production of autoantibodies targeting desmoglein 1 and/or 3, key components of desmosomal adhesion, leading to the loss of keratinocyte intercellular adhesion (1). Clinically, pemphigus can be categorized into two main types: pemphigus vulgaris (PV) and pemphigus foliaceus (PF) (2).

Systemic corticosteroids have long been the cornerstone of first-line therapy for pemphigus. However, the administration of high cumulative corticosteroids doses is associated with significant adverse events (AEs), including hyperglycemia, hypertension, dyslipidemia, osteoporosis, and gastrointestinal complications such as bleeding and ulceration (3). To mitigate these risks, several immunosuppressants, including azathioprine, mycophenolate mofetil, methotrexate, cyclophosphamide, cyclosporine, and dapsone, are often used as steroid-sparing agents to reduce complications (4). Recently, biological therapies have emerged as a promising therapeutic option, particularly for refractory or severe cases in which conventional treatments fail to achieve adequate disease control (DC) (3).

Rituximab (RTX), a chimeric murine/human anti-CD20 monoclonal antibody, has been approved for refractory pemphigus due to its targeted depletion of pathogenic B lymphocytes, offering a promising therapeutic option (5). The landmark Ritux 3 study demonstrated superior outcomes in moderate-to-severe PV patients treated with a combination of RTX and short-term prednisone compared to prednisone alone (6). Despite its established efficacy, no consensus exists regarding the optimal RTX dosing regimen for pemphigus. Various regimens have been adopted in clinical practice. The most commonly used protocols include the lymphoma protocol (four weekly infusions of 375 mg/m^2^ RTX), and the rheumatoid arthritis (RA) protocol (two infusions of 1000 mg RTX 14 days apart) (7).

Unlike lymphoma patients, pemphigus patients exhibit significantly lower B-cell burden. Emerging evidence indicates that reduced RTX doses can achieve therapeutic efficacy, with studies reporting complete remission (CR) after a single 250 mg RTX dose and sustained B-cell depletion for up to 6 months (8). Pharmacodynamic studies in healthy volunteers have demonstrated that a single ultra-low dose of RTX (1 mg/m^2^) effectively depletes CD20+ cells (9), supporting the biological plausibility of dose reduction strategies. Low-dose RTX regimens appear to be safer and more affordable, driving growing clinical interest in their application. A meta-analysis conducted in 2015 examined the efficacy of different RTX regimens for pemphigus patients and found no significant difference in CR or relapse rates between high-dose and low-dose RTX protocols (10). However, new studies have emerged over the past decade, highlighting the need for an updated evaluation of low-dose RTX in the treatment of pemphigus. This systematic review and meta-analysis aims to provide an updated evidence synthesis on the efficacy and safety of low-dose RTX in patients with pemphigus. We anticipate that our findings will contribute to evidence-based practice, potentially facilitating more personalized, cost-effective therapeutic approaches while maintaining clinical efficacy.

## 2 Materials and methods

### 2.1 Search strategy

We conducted a systematic review of the literature by searching the following databases: PubMed, Embase, the Cochrane Library, and ClinicalTrials.gov. The search spanned from the inception of each database to November 20, 2024, and was designed to identify eligible studies without restrictions on language, nationality, ethnicity, age, or cost. The search keywords were “low-dose RTX” and “pemphigus”, and the search strategy in PubMed was as follows: (“pemphigus” OR “pemphigus vulgaris” OR “pemphigus foliaceus”) AND (low-dose RTX).

### 2.2 Inclusion and exclusion criteria

Our inclusion criteria for studies in this analysis were as follows: (1) Both prospective and retrospective studies, including comparative and single-arm designs; (2) Studies involving patients diagnosed with pemphigus who were treated with low-dose RTX; (3) Studies providing data on efficacy and/or safety endpoints, including CR, relapse rates, time to disease control (TDC), time to CR, cumulative corticosteroid dose and AEs; (4) Patients enrolled in clinical studies.

Studies were excluded if they met any of the following criteria: (1) Sample cohort size (<5 patients); (2) Significant baseline disease severity disparities between groups in comparative studies prior to RTX treatment; (3) Enrollment of pediatric patients (age<18 years); (4) Patients with concurrent bullous pemphigoid (BP), mucous membrane pemphigoid (MMP), or other autoimmune bullous diseases (AIBDs), or those who transformed into BP, MMP, or other AIBDs; (5) Concurrent use of intravenous immunoglobulins with RTX; (6) Incomplete data or missing key efficacy/safety endpoints; (7) Non-original research (review, meta-analysis, case report) or preclinical studies (cell or animal-based research).

### 2.3 Quality assessment

Two investigators (SH L and SH W) independently conducted the quality assessment, with any discrepancies resolved through discussion and adjudication by a third author (YG Z). The quality of included randomized controlled trials was evaluated using the Modified Jadad scale (11), while the quality of non-randomized comparative studies and single-arm studies was assessed using the Methodological Index for Non-Randomized Studies (MINORS) (12). The Grading of Recommendations, Assessment, Development, and Evaluation (GRADE) approach (13) was used to evaluate the quality of evidence for our meta-analysis outcomes. All assessments were independently conducted by two investigators, followed by discussion until consensus was reached. Publication bias was evaluated by visual inspection of funnel plot asymmetry.

### 2.4 Data extraction

Study selection was independently performed by the first and second authors (SH L and SH W), with any discrepancies resolved by a third author (YG Z). The following details from the included studies were meticulously documented: authorship, publication year, country, sample size, gender, age, diagnosis, RTX therapeutic regimen, disease severity, follow-up duration, and endpoints. Efficacy outcomes (CR, relapse rates, TDC, time to CR, and cumulative corticosteroid dose) and safety outcomes (AEs) were captured in individually designed data sheets. Although Zhou X, et al. (14) reported outcomes for the low-dose RTX group, the comparative group did not receive high-dose RTX; therefore, it was classified as a single-arm study. In the study by Metta Parvathi et al. (15), which included both pemphigus and BP patients, data were extracted solely for the pemphigus patients. The study by Cho et al. (16) was treated as a single-arm study due to a significant difference in pemphigus severity between the high-dose and low-dose groups at baseline, rendering the comparative outcome unsuitable for our meta-analysis.

### 2.5 Definition

The definitions of DC, CR, and relapse were developed according to the consensus statement published in 2008 by the International Pemphigus Committee (17). DC was defined as the cessation of new lesion formation and the beginning of healing of established lesions. CR was defined as the absence of new or established lesions for at least two months. CR can be further subdivided into CR ON therapy and CR OFF therapy based on treatment status; however, these were not distinguished in this article. Relapse was defined as the appearance of three or more new lesions in a month that do not heal spontaneously within one week, or the extension of established lesions, in patients who previously achieved DC. Most included studies followed the definitions mentioned above, except for two studies. One study (18) didn’t provide a specific definition for CR in the original text, while another (19) defined relapse as CD19+ B-cell repopulation exceeding 1% of the total lymphocyte count (immunological relapse).

### 2.6 Statistical analysis

Statistical analysis was performed using Review Manager Version 5.4.1. For comparative studies, we calculated risk ratios (RR) with 95% confidence intervals (CIs) for dichotomous outcomes and the Standardized Mean difference (SMD) for continuous data. A two-tailed p value of < 0.05 was considered statistically significant. In single-arm studies, data were transformed from Odds Ratio to obtain pooled results. Heterogeneity between studies was assessed using the I^2^ statistic. For pooled findings with low heterogeneity (I^2^ < 50%), a fixed-effects model was employed; otherwise, a random-effects model was employed. In cases of high heterogeneity (I² ≥50%), sensitivity analysis was conducted by excluding each study to evaluate its impact on the pooled results.

## 3 Results

### 3.1 Study selection and characteristics

**Figure 1:**
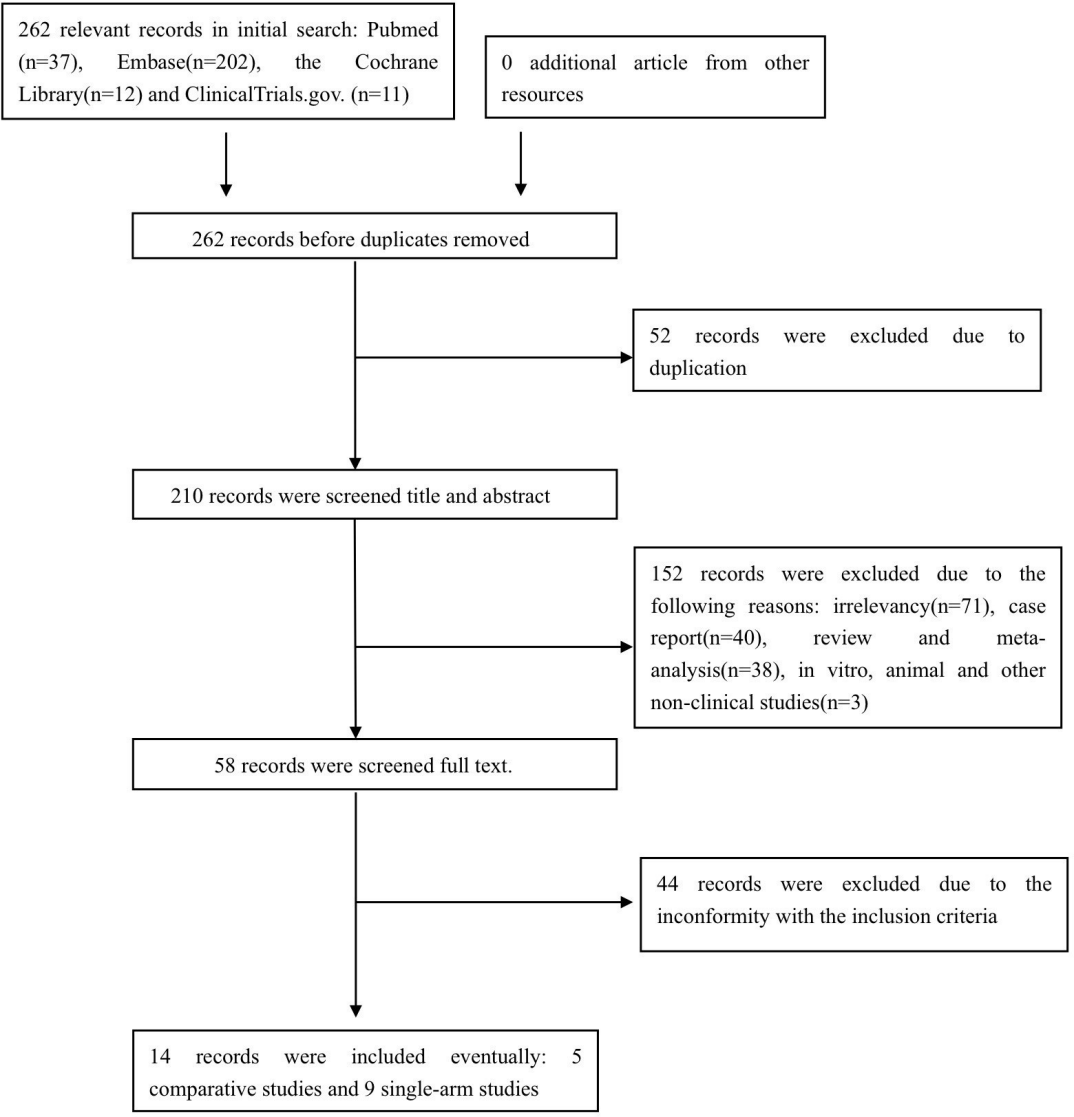
Flow chart of studies selection process.

The preliminary search identified pertinent references in PubMed (n =37), Embase (n = 202), the Cochrane Library (n = 12), and ClinicalTrials.gov (n = 11). After excluding duplicated, irrelevant, or ineligible studies, a total of 14 studies (14–16, 18–28) comprising 252 pemphigus patients were included in the final analysis, comprising nine prospective and five retrospective studies, with five comparative and nine single-arm study designs. The study selection process is illustrated in **Figure 1**.

The studies originated from various countries: four from India, three from China, two each from Australia and Korea, and one each from the Netherlands, Egypt and Italy. It is important to note that the definition of low-dose RTX used in this analysis is distinct from the traditional RA and lymphoma protocols, with the most frequently used regimen being two infusions of 500 mg (adopted in seven studies: 15, 19–24). Alternative RTX regimens included single infusions of 1000 mg (18), 500 mg (14), or 200 mg (25), two infusions of 375 mg/m^2^ (16, 26), and double infusions of 200 mg (27). Although the total dose of the four-infusion 500 mg regimen (28) is equivalent to that of the RA protocol, its reduced per-infusion dosage qualified it as low-dose RTX in our analysis. The characteristics of the included studies are detailed in **Table 1**.

**Table 1:** Characteristics of the included studies.

Study	Year	Country	Sample size	Gender male/female	Age (year)	Diagnosis	Severity	RTX^1^ regimen	FU^2^ (month)	Endpoints
Comparative studies										
(21)	2023	China	23 RTX:10 LRTX:13	13/10	RTX:42 ± 11 LRTX^3^:52 ± 15	RTX:9PV^4^ ,1PF^5^ LRTX:11PV,2PF	PDAI^6^ ≥25	RTX:1000 mg×2 LRTX:500 mg×2	12	CR^7^, Relapse, TDC^8^, Time to CR, Cumulative corticosteroid dose, AEs^9^
(19)	2022	India	23 RTX:12 LRTX:11	15/8	RTX:49.9 ± 10 LRTX:42.6 ± 12	21PV, 2PF	PDAI>25	RTX:1000 mg×2 LRTX:500 mg×2	12–20	CR, Relapse, TDC, Time to CR, Cumulative corticosteroid dose, AEs
(18)	2020	Egypt	14 RTX:7 LRTX:7	5/9	34-65	NA^10^	PDAI 2-17	RTX:1000 mg×2 LRTX:100 mg×1	4	CR
(23)	2014	India	22 RTX:11 LRTX:11	11/11	RTX: 33.18 ± 9.94 LRTX: 33.55 ± 13.07	RTX:7PV,4 PF LRTX:8PV,3PF	Ikeda severity score:RTX: 6.36 LRTX:5.91 Mucosal: RTX:4.55 LRTX:2.36	RTX:1000 mg×2 LRTX:500 mg×2	12	CR, Relapse, TDC, Time to CR, Cumulative corticosteroid dose, AEs
(26)	2011	Korea	27 LRTX:12 RTX:15	14/13	LRTX: 48.4 ± 10.1 RTX: 47.7 ± 14.3	25PV,2PF	Pemphigus severity score: RTX:9-13 LRTX:9-12	LRTX:375 mg/m^2^×2 RTX:375 mg/m^2^× 3-5	LRTX:11.5 RTX:18.0	CR, Relapse, TDC, Time to CR, AEs
Single-arm studies										
(14)	2024	China	19	6/13	44.21 ± 12.59	19PV	PDAI:22.50(32.00)	500 mg×1	12	CR, AEs
(15)	2024	India	20	NA	Pemphigus:53 BP:64	9PV,3PF,8 BP^11^	ABSIS^12^ : 54-80	500 mg×2	12	CR, AEs
(27)	2022	Australia	8	NA	NA	NA	NA	200 mg×2	NA	CR, Relapse, AEs
(25)	2020	Italy	8	5/3	34-73	8PV	PDAI>25	200 mg×1	4.5-25.3	CR, Relapse, AEs
(22)	2018	Australia	9	3/6	35-75	8PV,1PF	NA	500 mg ×2	1-38.5	CR, Relapse, AEs
(20)	2017	India	50	20/30	9-65	41PV,9PF	PAS^13^:3-9	500 mg×2	12-25	CR, Relapse, AEs
(28)	2014	China	9	5/4	36-79	6PV,3PF	Moderate-severe	500 mg×4	15.3-32.3	CR, Relapse, AEs
(24)	2012	Netherlands	15	10/5	34-80	12PV,3PF	NA	500 mg×2	8-38	CR, Relapse, AEs
(16)	2014	Korea	13	6/7	31-80	8PV,5PF	PAS<9	375 mg/m^2^×2	17.8	CR, Relapse, AEs

*The data in the table are presented as range/median (IQR)/mean/mean ± SD.

^1^Rituximab, ^2^Follow-up, ^3^Low-dose rituximab, ^4^Pemphigus vulgaris, ^5^Pemphigus foliaceus, ^6^Pemphigus disease activity index, ^7^Complete remission, ^8^Time to disease control, ^9^Adverse effects, ^10^Not available, ^11^Bullous pemphigoid, ^12^Autoimmune Bullous Skin Disorder Intensity Score, ^13^Pemphigus activity score.

### 3.2 Quality assessment

Eleven studies (14–16, 20–22, 24–28) were evaluated using MINORS, with scores ranging from 11 to 14. These scores indicate satisfactory methodological quality, meeting the threshold for inclusion in this meta-analysis. Three randomized controlled trials (18, 19, 23) were assessed using the Modified Jadad scale, yielding scores of 4, 5, and 6, respectively, reflecting high quality methodology. Comprehensive quality assessment details are presented in **Supplementary Table S1**. The quality of evidence for our meta-analysis outcomes was evaluated and is presented in **Supplementary Table S2**. These results demonstrate high confidence in the findings on TDC, moderate confidence in the results on CR rate, time to CR, and cumulative corticosteroid dose, and limited confidence in the results on relapse rate. The funnel plots are presented in **Supplementary Figures S1**-**S3**.

### 3.3 Efficacy

#### 3.3.1 CR

**Figure 2:**
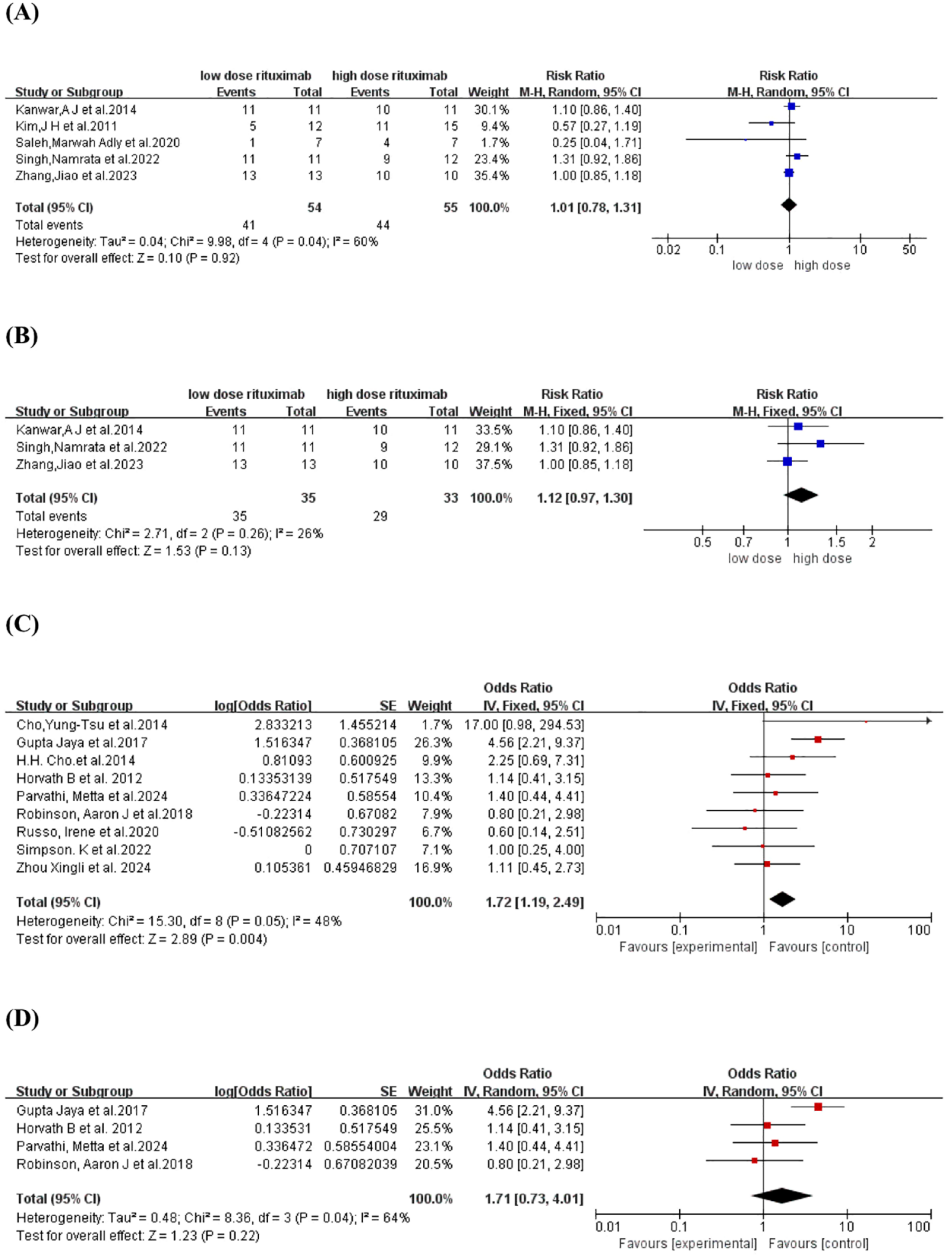
Forest plot of CR rate. **(A)** Five comparative studies **(B)** Three comparative studies comparing 2 infusions of 1000mg versus 500mg RTX **(C)** Nine single-arm studies **(D)** Four single-arm studies using two infusions of 500mg RTX.

The CR rate was reported in five comparative studies (18, 19, 21, 23, 26) which included a total of 109 patients treated with low-dose (n=54) and high-dose RTX (n=55). Pooled analyses from comparative studies revealed no significant difference in CR rate between the low-dose and high-dose RTX groups (RR, 1.01, 95% CI: 0.78 to 1.31, *I^2^* = 60%, *P* = 0.92) (**Figure 2A**). Further comparison of the CR rate between the subgroups receiving two infusions of 1000 mg and 500 mg RTX (19, 21, 23) also showed no significant difference (RR:1.12, 95% CI: 0.97 to 1.30, *I^2^* = 26%, *P* = 0.13) (**Figure 2B**). In the pooled analysis of nine single-arm studies (14–16, 20, 22, 24, 25, 27, 28), the overall CR rate was 63.2% (95% CI: 54.3% to 71.3%, *I^2^* = 48%, *P* = 0.04) (**Figure 2C**). Among these, four single-arm studies (15, 20, 22, 24) utilized the protocol of two infusions of 500 mg RTX, with a pooled CR rate of 63.1% (95% CI: 42.2% to 80.0%, *I^2^* = 64%, *P* = 0.22) (**Figure 2D**).

#### 3.3.2 Relapse

**Figure 3:**
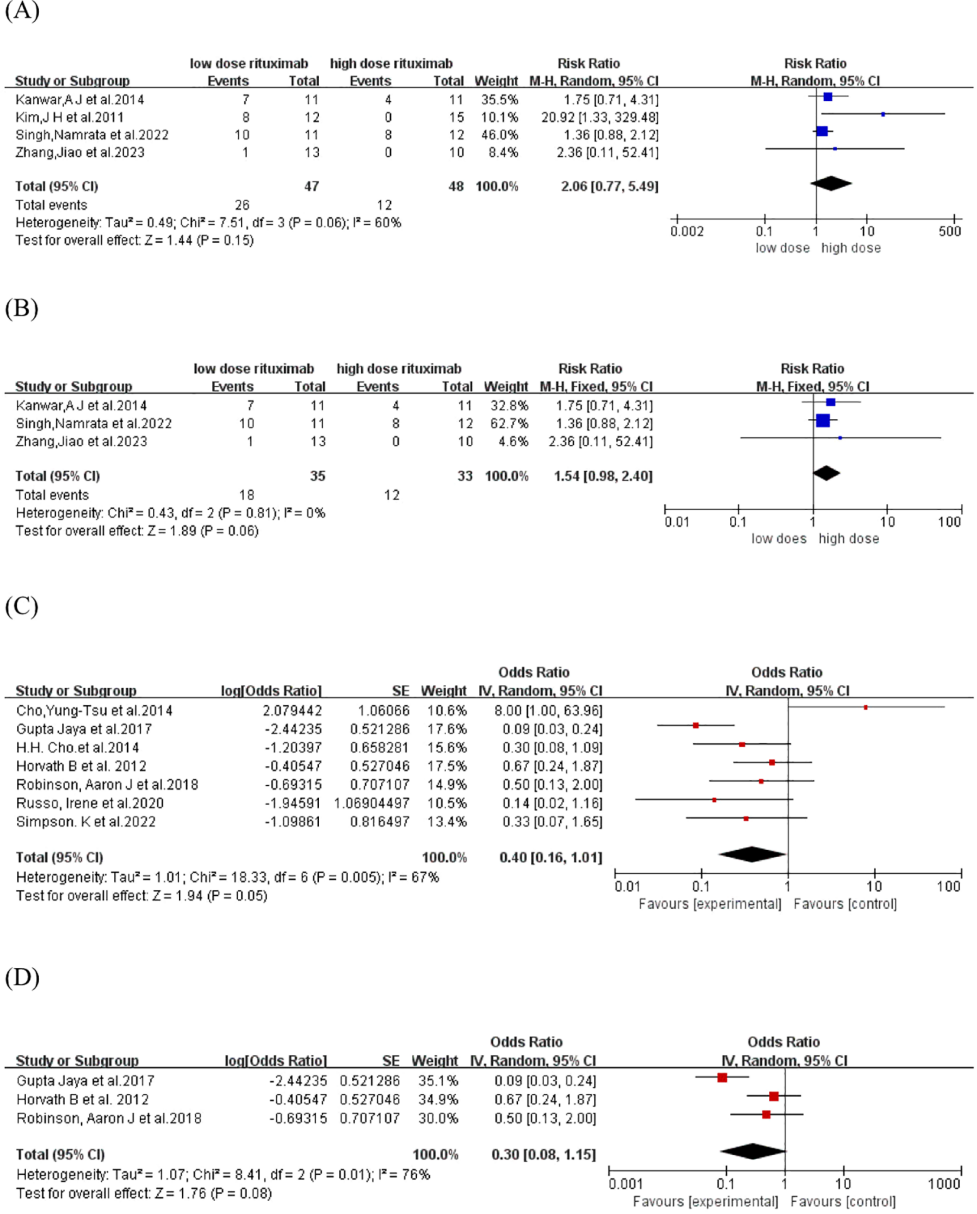
Forest plot of relapse rate. **(A)** Four comparative studies **(B)**Three comparative studies comparing 2 infusions of 1000mg versus 500mg RTX **(C)** Seven single-arm studies **(D)** Three single-arm studies using two infusions of 500mg RTX.

The relapse rate was reported in four comparative studies (19, 21, 23, 26), which included a total of 95 patients treated with low-dose (n=47) and high-dose RTX (n=48). Pooled analyses from these comparative studies revealed no significant difference in the relapse rate between low-dose and high-dose RTX groups (RR: 2.06, 95% CI: 0.77 to 5.49, *I^2^* = 60%, *P* =0.15) (**Figure 3A**). Further comparison of the relapse rate between the subgroups receiving two infusions of 1000 mg and 500 mg RTX (19, 21, 23) also showed no significant difference (RR: 1.54, 95% CI: 0.98 to 2.40, *I^2^* = 0%, *P* = 0.06) (**Figure 3B**). In the pooled analysis of seven single-arm studies (16, 20, 22, 24, 25, 27, 28), the overall relapse rate was 28.6% (95% CI: 13.8% to 50.2%, *I^2^* = 67%, *P* = 0.05) (**Figure 3C**). Among these, three single-arm studies (20, 22, 24) utilized the protocol of two infusions of 500 mg RTX, with a pooled relapse rate of 23.1% (95% CI: 7.4% to 53.5%, *I^2^* = 76%, *P* = 0.08) (**Figure 3D**).

#### 3.3.3 TDC, time to CR

**Figure 4:**
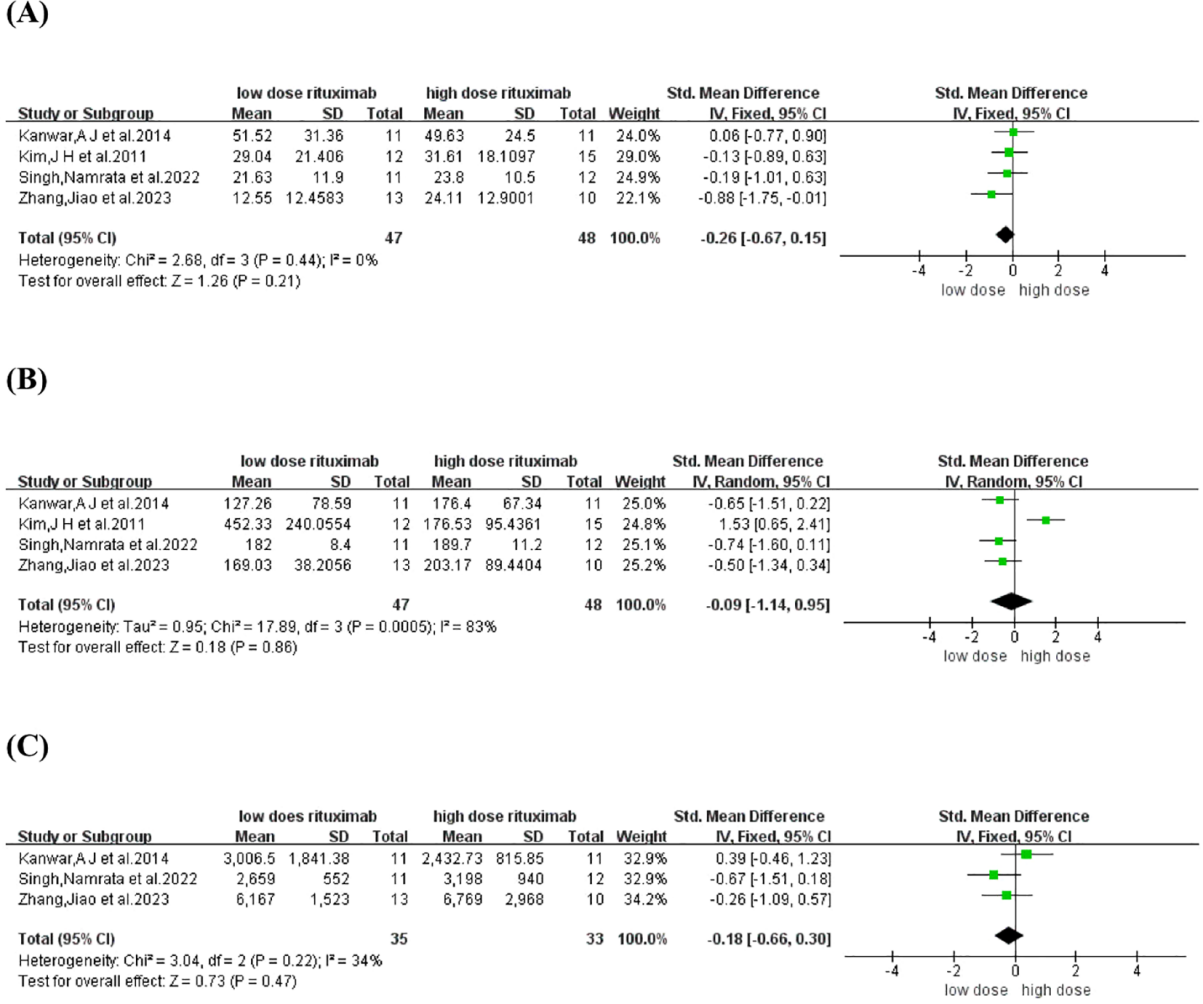
Forest plot of **(A)** time to disease control **(B)** time to CR **(C)** Cumulative corticosteroid dose.

TDC and time to CR were reported in four comparative studies (19, 21, 23, 26), which included 95 patients treated with low-dose RTX (n=47) and high-dose RTX (n=48). Pooled analyses from comparative studies revealed no significant difference in TDC or time to CR between the low-dose and high-dose RTX groups (SMD: -0.26, -0.09, 95% CI: -0.67 to 0.15, -1.14 to 0.95, *I^2^* = 0%, 83%, *P* = 0.21, 0.86) (**Figures 4A, B**).

#### 3.3.4 Cumulative corticosteroid dose

Cumulative corticosteroid dose was reported in three comparative studies (19, 21, 23), which included 68 patients treated with low-dose (n=35) and high-dose RTX (n=33). Pooled analyses from these comparative studies revealed no significant difference in cumulative corticosteroid dose between low-dose and high-dose RTX groups (SMD: -0.18, 95% CI: -0.66 to 0.30, *I^2^* = 34%, *P* = 0.47) (**Figure 4C**).

### 3.4 Safety

AEs related to RTX infusion were reported in thirteen studies. Two deaths due to pneumonia and sepsis occurred in patients who received two infusions of 1000 mg RTX. Additionally, three patients who received either a single infusion of 200 mg RTX or two infusions of 500 mg RTX survived episodes of pneumonia and sepsis. No deaths were reported in the low-dose RTX group. The most common AEs were infections, including viral, bacterial, and fungal. Infusion-related reactions, including transient fever, tachycardia, chills, hypotension, and urticaria, were also observed, emphasizing the need for close monitoring during administration. However, attributing all AEs solely to RTX remains challenging due to the concurrent use of other medications, such as corticosteroids and immunosuppressants. The overlapping AE profiles complicate the identification of the primary causative agent. A summary of the reported AEs and concomitant medications from each included study is presented in **Table 2**.

**Table 2:** Reported AEs in included studies.

Study	RTX^1^ regimen	Contaminant therapy	AEs^2^
(21)	LRTX^3^:500 mg×2 RTX:1000 mg×2	LRTX: corticosteroids (13/13), immunosuppressive treatment (0/13) RTX: corticosteroids (10/10), immunosuppressive treatment (0/10)	No severe AEs
(19)	LRTX:500 mg×2 RTX:1000 mg×2	LRTX: corticosteroids (11/11), immunosuppressive treatment (0/11) RTX: corticosteroids (12/12), immunosuppressive treatment (0/12)	LRTX: Furunculosis (3), Urinary tract infection (3), Dermatophytosis (2), Oral candidiasis, Herpes labialis, Scabies RTX: Furunculosis (3), Oral candidiasis, Genital wart, Cataract, Glaucoma, Diabetes (new onset), Osteoporosis, Supraventricular tachycardia, Pneumonia (death), Septicemia (death)
(16)	LRTX:375 mg/m^2^×2 RTX:375 mg/m^2^×3-4	LRTX: corticosteroids (13/13), immunosuppressive treatment (6/13) RTX: corticosteroids (10/10), immunosuppressive treatment (10/10)	Mild, transient fever and tachycardia during infusion
(23)	LRTX:500 mg×2 RTX:1000 mg×2	LRTX: corticosteroids (10/11), immunosuppressive treatment (5/11) RTX: corticosteroids (11/11), immunosuppressive treatment (2/11)	LRTX and RTX: Upper respiratory tract infections, Diarrhea, Striae, Acneiform eruptions. No major AEs were seen in either group.
(26)	LRTX: 375 mg/m^2^×2 RTX:375 mg/m^2^×3-5	NA^4^	No severe AEs
(14)	500 mg×1	corticosteroids (19/19)	Elevation of liver enzyme (3), Folliculitis, Metabolic abnormalities (hyperlipidemia, hypokalemia, diabetes mellitus), Hypertension, Viral infection
(15)	500 mg×2	NA	Chills and hypotension following infusion, Flu-like symptoms (2) and Herpes Zoster during follow-up
(27)	200 mg×2	corticosteroids	No severe AEs
(25)	200 mg×1	corticosteroids (8/8), immunosuppressive treatment (0/8)	Sepsis due to Citrobacer freundii infection, Pneumonia due to Haemophilus influenzae, and other patients didn’t experience any AEs.
(22)	500 mg×2	corticosteroids (7/9), immunosuppressive treatment (9/9), IVIG (1/9)	Cutaneous infection (5 in 2 patients), Lymphopenia (2), Herpes simplex virus, Gastro-esophageal reflux disease, Non-alcoholic fatty liver disease, Osteoporosis, Upper respiratory tract infection, Oral candidiasis, HSV supraglottitis, Trochanteric bursitis, Cytomegalovirus, Balanitis, Tinea pedis
(20)	500 mg×2	NA	Chills and urticaria following infusion, Decreased blood pressure and Herpes zoster
(28)	500 mg×4	corticosteroids (9/9)	No complications
(24)	500 mg×2	corticosteroids (9/15), immunosuppressive treatment (11/15)	Mild, early AEs: Influenza-like symptoms (2), Mild herpes zoster, Atrioventricular nodal reentry tachycardia with chest pain (arrhythmias in his medical history) Serious AEs: Sepsis

^1^Rituximab, ^2^adverse events, ^3^low-dose rituximab, ^4^NA, Not available.

## 4 Discussion

RTX has become increasingly pivotal in the treatment of pemphigus, though the optimal dosing regimen has not been fully established. Our meta-analysis demonstrated comparable clinical efficacy between low-dose and high-dose RTX regimens, with no statistically significant differences in CR or relapse rates, consistent with previous findings (10). Moreover, we innovatively demonstrated that the low-dose RTX cohort did not prolong TDC or time to CR, nor did it increase the overall cumulative corticosteroid dose compared to high-dose RTX. The pooled CR and relapse rates in single-arm studies were 63.2% and 28.6%, respectively. Importantly, while severe infections were documented in the low-dose RTX group, no treatment-related mortality was observed, supporting its overall safety profile when administered with appropriate monitoring.

Beyond pemphigus, low-dose RTX has been used to treat various autoimmune blistering diseases, including BP, MMP, epidermolysis bullosa acquisita, and linear IgA disease (29–32). Ultra-low dose RTX protocols have also been explored for pemphigus treatment. For instance, two PV patients were successfully treated with a single intravenous infusion of 200 mg RTX (33). A case report from China described a refractory PV patient who achieved total clearance of skin lesions three months after a single infusion of 100 mg RTX (34). Additionally, a large case series involving 16 PV patients reported a satisfactory CR rate (93.75%) after two infusions of 100 mg RTX (35). However, these preliminary findings require validation through controlled trials. Ongoing studies are exploring optimized administration schedules, such as a modified lymphoma protocol with extended infusion intervals, which has shown favorable outcomes with reduced side effects (36).

Relapse remains a common and challenging issue in clinical practice, imposing significant emotional and economic burden on patients. Mucosal involvement (37), anti-desmoglein3 IgG subclasses (particularly IgG3) (38), and delayed RTX initiation (39, 40) have been identified as key predictors of disease relapse in pemphigus patients treated with RTX.

Our study advances current understanding by incorporating novel single-arm meta-analyses and subgroup analyses while confirming previous conclusions (10). However, we acknowledge several limitations in our study. First, due to the rarity of pemphigus, only 14 articles comprising 252 patients were included in this meta-analysis. Second, the accuracy of the pooled outcomes may be affected by differences in disease severity, treatment regimens, and follow-up duration. Third, we were unable to directly compare the incidence of AEs between the low-dose and high-dose RTX groups due to insufficient detailed information in the original studies.

In the future, we anticipate more prospective, large-scale, randomized controlled trials to definitively establish dose-response relationships and compare protocol efficacy (RA vs. lymphoma regimens). Such efforts will facilitate the development of personalized, cost-effective treatment algorithms that maximize therapeutic outcomes while minimizing both clinical and economic burdens for pemphigus patients worldwide.
