# Supplementary material for: Efficacy and safety of low dose rituximab in pemphigus: an updated systematic review and meta-analysis
Source: Front Immunol. 2025 Jul 25;16:1605243. doi: 10.3389/fimmu.2025.1605243 (PMC12331757; doi:10.3389/fimmu.2025.1605243)
Supplement: Supplementary file 1 [file Table1.docx]

Supplementary Material

1. **Supplementary Figures and Tables**

## Supplementary Figures


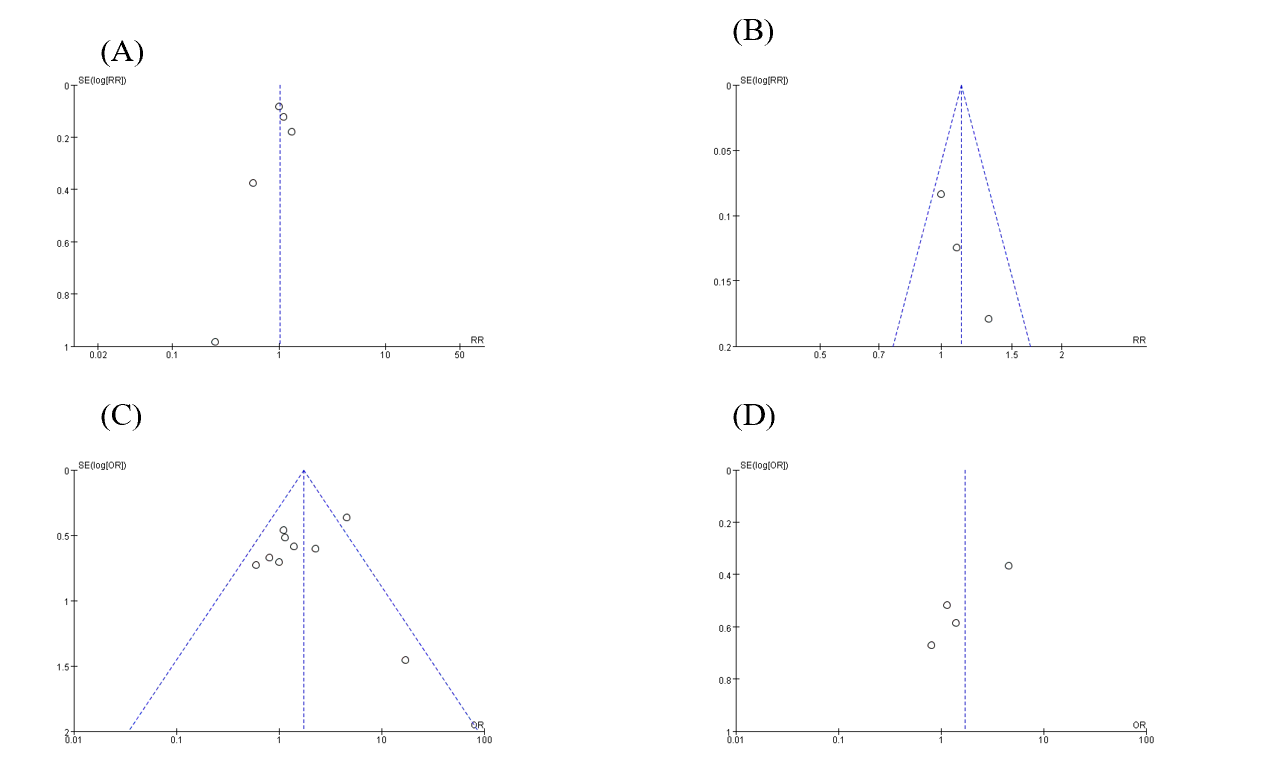


Fig S1. Funnel plot of CR rate. (A)Five comparative studies (B)Three comparative studies comparing two infusions of 1000mg versus 500mg RTX (C)Nine single-arm studies (D)Four single-arm studies using two infusions of 500mg RTX.


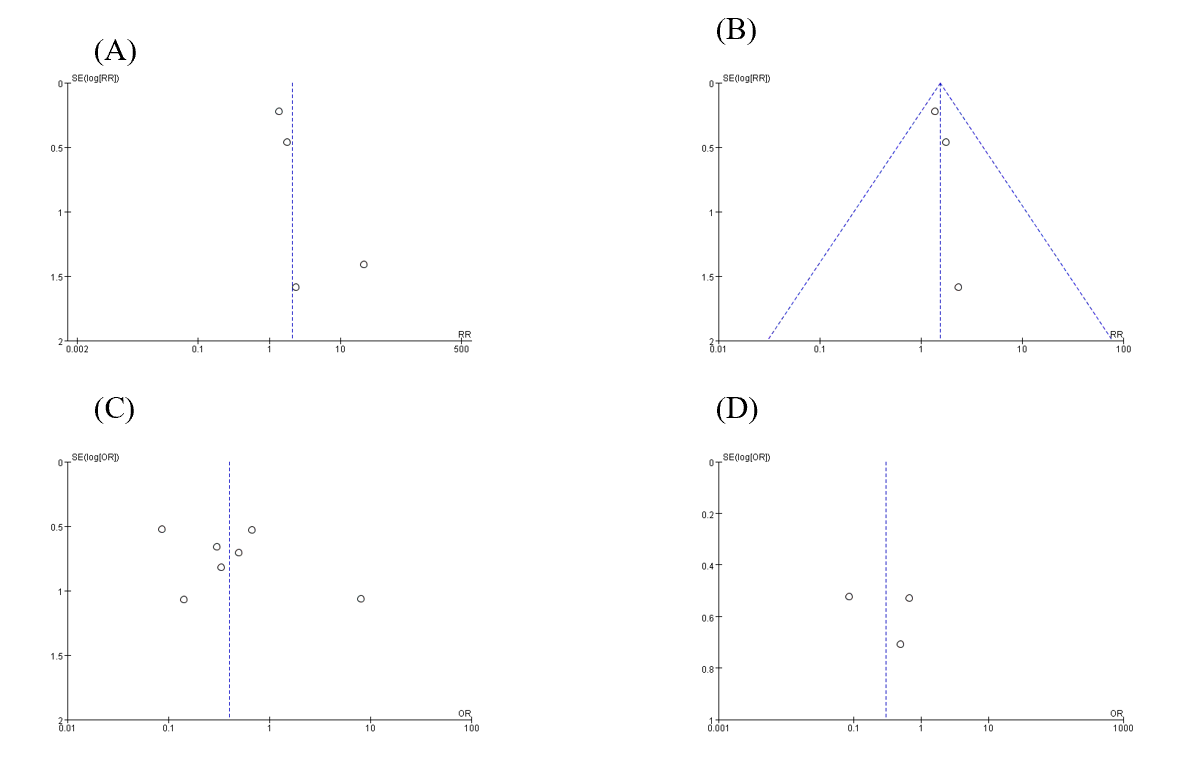


Fig S2. Funnel plot of relapse rate. (A)Four comparative studies (B)Three comparative studies comparing two infusions of 1000mg versus 500mg RTX (C)Seven single-arm studies (D)Three single-arm studies using two infusions of 500mg RTX.


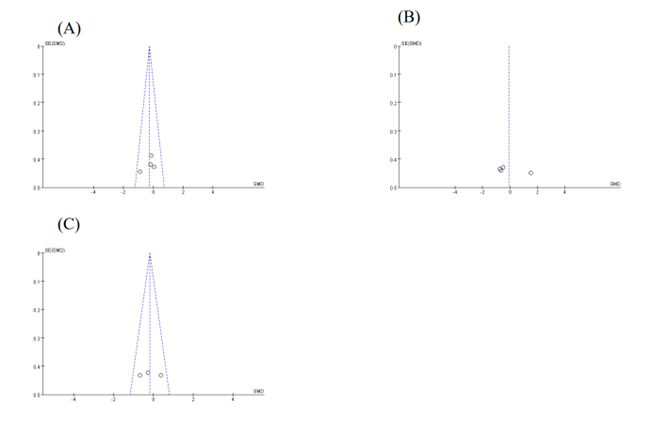


Fig S3. Funnel plot of (A)Time to disease control (B)Time to CR (C) Cumulative corticosteroid dose.

## Supplementary Tables

Table S1. Quality assessment of included studies.

| **MINORS** | I^[[1]](#footnote-1)^ | II^[[2]](#footnote-2)^ | III^[[3]](#footnote-3)^ | IV^[[4]](#footnote-4)^ | V^[[5]](#footnote-5)^ | VI^[[6]](#footnote-6)^ | VII^[[7]](#footnote-7)^ | VIII^[[8]](#footnote-8)^ | Total |  |
| --- | --- | --- | --- | --- | --- | --- | --- | --- | --- | --- |
| Zhou X, et al. (14) | 2 | 2 | 2 | 2 | 2 | 2 | 2 | 0 | 14 |  |
| Zhang, Jiao et al. (21) | 2 | 1 | 2 | 2 | 2 | 2 | 2 | 0 | 13 |  |
| Metta Parvathi et al. (15) | 2 | 2 | 2 | 2 | 2 | 2 | 2 | 0 | 14 |  |
| Jaya Gupta et al. (20) | 2 | 2 | 2 | 2 | 2 | 2 | 2 | 0 | 14 |  |
| B. Horva´th, J.et al.(24) | 2 | 1 | 2 | 2 | 2 | 2 | 2 | 0 | 13 |  |
| Irene Russo et al. (25) | 2 | 1 | 2 | 2 | 2 | 2 | 2 | 0 | 13 |  |
| H.H. Cho.et al. (16) | 2 | 1 | 0 | 2 | 2 | 2 | 2 | 0 | 11 |  |
| Aaron J Robinson et al. (22) | 2 | 1 | 0 | 2 | 2 | 2 | 2 | 0 | 11 |  |
| J.H. Kim et al. (26) | 2 | 1 | 0 | 2 | 2 | 2 | 2 | 0 | 11 |  |
| Simpson. K et al. (27) | 2 | 1 | 0 | 2 | 2 | 2 | 2 | 0 | 11 |  |
| Cho, Yung-Tsu et al. (28) | 2 | 1 | 0 | 2 | 2 | 2 | 2 | 0 | 11 |  |
| **Modified Jadad Scale** | Randomization | | Concealment of allocation | | Double blinding | | Withdrawals and dropouts | | Total | |
| Namrata Singh et al. (19) | 2 | | 1 | | 0 | | 1 | | 4 | |
| A.J. Kanwar et al. (23) | 2 | | 1 | | 1 | | 1 | | 5 | |
| Marwah Adly Saleh et al. (18) | 2 | | 1 | | 2 | | 1 | | 6 | |

Table S2. Quality of evidence using Grade of Recommendations Assessment, Development and Evaluation (GRADE) System.

| **Number of studies** | **Study design**^[[9]](#footnote-9)^ | **Certainty assessment** | | | | | | **Number of patients** | | **Effect**  **RR/SMD**  **(95% CI)** | **Certainty** |
| --- | --- | --- | --- | --- | --- | --- | --- | --- | --- | --- | --- |
|  |  | **Risk of bias**^[[10]](#footnote-10)^ | **Inconsistency**^[[11]](#footnote-11)^ | **Indirectness** | **Imprecision**^[[12]](#footnote-12)^ | **Publication bias**^[[13]](#footnote-13)^ | **Other considerations** | **Low-dose rituximab** | **High-dose rituximab** |  |  |
| **CR rate** | | | | | | | | | | | |
| 5 | Randomized trials  (3)  Observational (2) | Not serious | Serious | Not serious | Not serious | Not serious | None | 54 | 55 | RR 1.01(0.78 to 1.31) | ⨁⨁⨁◯ Moderate |
| 3 | Randomized trials  (2)  Observational (1) | Not serious | Serious | Not serious | Not serious | Not serious | None | 35 | 33 | RR 1.12(0.97 to 1.30) | ⨁⨁⨁◯ Moderate |
| **Relapse rate** | | | | | | | | | | | |
| 4 | Randomized trials  (2)  Observational (2) | Not serious | Serious | Not serious | Serious | Not serious | None | 47 | 48 | RR 2.06(0.77 to 5.49) | ⨁⨁◯◯ Low |
| 3 | Randomized trials  (2)  Observational (1) | Not serious | Not serious | Not serious | Serious | Not serious | None | 35 | 33 | RR 1.54(0.98 to 2.40) | ⨁⨁⨁◯ Moderate |
| **Time to disease control** | | | | | | | | | | | |
| 4 | Randomized trials  (2)  Observational (2) | Not serious | Not serious | Not serious | Not serious | Not serious | None | 47 | 48 | SMD-0.26(-0.67-0.15) | ⨁⨁⨁⨁  High |
| **Time to CR** | | | | | | | | | | | |
| 4 | Randomized trials  (2)  Observational (2) | Not serious | Serious | Not serious | Not serious | Not serious | None | 47 | 48 | SMD-0.09(-1.14-0.95) | ⨁⨁⨁◯ Moderate |
| **Cumulative corticosteroid dose** | | | | | | | | | | | |
| 3 | Randomized trials  (2)  Observational (1) | Not serious | Not serious | Not serious | Serious | Not serious | None | 35 | 33 | SMD-0.18(-0.66-0.30) | ⨁⨁⨁◯ Moderate |

1. a clearly stated aim [↑](#footnote-ref-1)
2. inclusion of consecutive patients [↑](#footnote-ref-2)
3. prospective collection of data [↑](#footnote-ref-3)
4. endpoints appropriate to the aim of the study [↑](#footnote-ref-4)
5. unbiased assessment of the study endpoint [↑](#footnote-ref-5)
6. follow-up period appropriate to the aim of the study [↑](#footnote-ref-6)
7. loss of follow up less than 5% [↑](#footnote-ref-7)
8. prospective calculation of the study size [↑](#footnote-ref-8)
9. The initial quality is set as high as all observational studies included have comparable diseases severity at baseline. [↑](#footnote-ref-9)
10. Downgrading one level for the inadequate randomization and high dropout rates. [↑](#footnote-ref-10)
11. Downgrading one level for the heterogeneity ≥ 50%. [↑](#footnote-ref-11)
12. Downgrading one level for small sample size and wide confidence intervals. [↑](#footnote-ref-12)
13. Downgrading one level for the visual asymmetry of funnel plot. [↑](#footnote-ref-13)
